# Optical Imaging of the Motor Cortex Following Antidromic Activation of the Corticospinal Tract after Spinal Cord Injury

**DOI:** 10.3389/fnins.2017.00166

**Published:** 2017-03-29

**Authors:** Kyung H. Lee, Un J. Kim, Se W. Park, Yong G. Park, Bae H. Lee

**Affiliations:** ^1^Division of Health Sciences, Department of Dental Hygiene, Dongseo UniversityBusan, South Korea; ^2^Department of Physiology, Yonsei University College of MedicineSeoul, South Korea; ^3^Ernest Mario School of Pharmacy, Rutgers UniversityNew Brunswick, NY, USA; ^4^Department of Neurosurgery, Yonsei University College of MedicineSeoul, South Korea; ^5^Brain Korea PLUS Project for Medical Science, Yonsei University College of MedicineSeoul, South Korea

**Keywords:** spinal cord injury, neuroplasticity, motor evoked potential, optical recording, electrophysiology

## Abstract

Spinal cord injury (SCI) disrupts neuronal networks of ascending and descending tracts at the site of injury, leading to a loss of motor function. Restoration and new circuit formation are important components of the recovery process, which involves collateral sprouting of injured and uninjured fibers. The present study was conducted to determine cortical responses to antidromic stimulation of the corticospinal tracts, to compare changes in the reorganization of neural pathways within normal and spinal cord-injured rats, and to elucidate differences in spatiotemporal activity patterns of the natural progression and reorganization of neural pathways in normal and SCI animals using optical imaging. Optical signals were recorded from the motor cortex in response to electrical stimulation of the ventral horn of the L1 spinal cord. Motor evoked potentials (MEPs) were evaluated to demonstrate endogenous recovery of physiological functions after SCI. A significantly shorter N1 peak latency and broader activation in the MEP optical recordings were observed at 4 weeks after SCI, compared to 1 week after SCI. Spatiotemporal patterns in the cerebral cortex differed depending on functional recovery. In the present study, optical imaging was found to be useful in revealing functional changes and may reflect conditions of reorganization and/or changes in surviving neurons after SCI.

## Introduction

Spinal cord injury (SCI) is a debilitating and devastating condition for most mammalian organisms. In the central nervous system (CNS), neuroanatomical reorganization reflects functional changes and plays a critical role in rehabilitation and functional recovery (Ghosh et al., 2010; Isa and Nishimura, 2014). Specifically, SCI causes disruption of networks of damaged axons and neural pathways, which is followed by restoration of plasticity and reorganization of neural networks in the spinal cord (Onifer et al., 2011). Neuroplasticity is the dynamic potential of the CNS to reorganize neural pathways and/or rehabilitate neuronal circuits (Nardone et al., 2013). Plasticity can be measured via the electrical activity of specific neurons: for instance, SCI patients showed plasticity in the somatosensory cortex and corticospinal tract that increased neuronal sensitivity (Bazley et al., 2014; Chisholm et al., 2015). Electrophysiology studies have shown evidence of network plasticity, such as endogenous compensation and recovery mechanisms of the spinal cord and brain, after SCI (Nardone et al., 2013). After SCI, new synapse formation and rearrangement occur in the cortical and subcortical areas. Surviving neurons also adapt through collateral sprouting of spared axons across the injury site (Ghosh et al., 2010; Silva et al., 2014). Notwithstanding, the mechanisms of plastic restoration and neural reorganization in functional adaptation after SCI remained unclear.

Electrophysiological evaluations of somatosensory evoked potentials (SSEPs) and motor evoked potentials (MEPs) provide an objective assessment of spinal cord conduction after SCI (Lee et al., 2005). MEPs reflect centripetal conduction of the motor pathway induced by stimulation of the motor conduction route. The amplitudes of MEPs have been shown to be correlated with amounts of spared/regenerated tissue (Nashmi et al., 1997; Garcia-Alias et al., 2003). Meanwhile, optical image recording with voltage-sensitive dyes (VSD) can be used to highlight the spatiotemporal distribution of neural activities and differences in the sensory network of the brain (Lee et al., 2011). While microelectrode arrays and single electrode recordings are limited in visualizing mass-activation of millions of neurons at different points, which would facilitate better understanding of large-scale cortical functional organization, optical imaging of cortical responses may be useful for the direct assessment of a cortical functional with sufficient spatial and temporal resolution that can be tracked in real-time.

This study was conducted to determine cortical responses to the stimulation of the corticospinal tract, and to compare changes in the reorganization of neural pathways in normal and spinal cord-injured rats. Additionally, we sought to elucidate differences in spatiotemporal activity patterns during natural progression and reorganization of neural pathways in normal and SCI animals using optical imaging methods.

## Methods

### Spinal cord injury

Forty Sprague-Dawley rats (300–350 g; Koatech, Pyeongtaek, Korea) were used as the SCI model. Rats were prepared by laminectomy of the thoracic 9 (T9) vertebrae and exposure of the dorsal spinal column surface. SCI was induced using a New York University, Multicenter Animal SCI Study (NYU MASCIS) weight-drop device. A 10-g weight impact rod was dropped from a height of 25 mm to produce a moderate contusion of the SC. An antibiotic (gentamycin sulfate; 1 mg/kg) was administered daily for 1 week.

All animals were housed in a facility accredited by the Association for Assessment and Accreditation of Laboratory Animal Care (AAALAC) and given food and water *ad libitum*, with alternating 12-h light/dark cycles. Animal experiments were approved by the Institutional Animal Care and Use Committee of the Yonsei University Health System (Permit No: 09-118). Animal sample sizes were kept to a minimum, and procedures were performed in a manner that minimized the potential for pain or distress to the animals.

### Behavioral test

The Basso, Beattie, and Bresnahan (BBB) test was performed to measure functional impairment or recovery of hind limbs (Basso et al., 1996). Forty rats were tested. Briefly, rats were gently adapted to an open field, and their behavior was observed once the rats were able to walk continuously. Two examiners conducted a 5-min preoperative test using the BBB locomotor rating scale in a double-blind design. Postoperative (p.o.) open field testing was conducted at 1, 3, 7, 14, 21, and 28 days p.o. for all animals.

### Optical imaging of antidromic activation of the corticospinal tract

Optical recording was performed at 1 and 4 weeks after SCI (1-W and 4-W SCI). We used age-matched rats as a control for each optical imaging recording time (1-W Control and 4-W Control). Forty male Sprague-Dawley rats were anesthetized with urethane (1.25 g/kg, i.p.) and mounted on a stereotaxic apparatus (Narishige Scientific Instrument Laboratory, Tokyo, Japan). A craniotomy was performed on the motor cortex, and the dura was resected. The hydrophilic voltage-sensitive dye (VSD) di-2 ANEPEQ (50 μg/mL in saline, Molecular Probes, Eugene, OR, USA) was applied directly into the exposed cortex and allowed to stain for 1 h. After staining, the cortex was kept moist with saline. During the procedure, the animal was artificially ventilated using an animal respirator (Model 683, Rodent Ventilator, Harvard Apparatus, Holliston, MA, USA). Vecuronium bromide (0.2 mg/kg, i.v., Huons Co., Hwaseong, Korea) was injected to eliminate interference by respiratory movements during optical measurement.

Optical signals were recorded from the motor cortex, the origin of the corticospinal tract, activated antidromically by stimulation of the contralateral gray matter (ventral horn) in the L1 spinal cord with a concentric bipolar electrode. Electrical stimulation (100 μs pulses, 0.1–9 mA intensity) was delivered via the bipolar stimulating electrode. Latencies were analyzed upon electrical stimulation with 6 mA. MEP latencies were classified as initial, N1, and P1 by observation. The initial latency was defined as the time interval between electrical stimulation and an initial rising phase of the MEP. N1 latency was defined as the time interval between electrical stimulation and the first negative (upward) peak. The P1 peak was ignored because of its inconsistent pattern.

The axis of the camera was positioned perpendicular to the surface of the brain and displayed the motor cortex in the image field. A change in fluorescence of the VSD (di-2 ANEPEQ) facilitated visualization of neuronal activity using an optical imaging recording system (MiCAM02, Brain Vision Inc., Tsukuba, Japan). A high resolution CCD camera (3.7 ms per frame maximum time) was utilized to detect differences in fluorescence. According to other experiments (Potts and Paton, 2006), optical imaging of eupnea typically requires 10 acquisition sweeps, while imaging of gasping requires only a single acquisition sweep. Image acquisition was triggered by an electrocardiogram using a stimulus/non-stimulus subtraction method (Orbach et al., 1985; Takashima et al., 2001). Magnification was provided by a 1 × objective and a 0.63 × projection lens (Leica Microsystems Ltd., Wetzlar, Germany), which yielded a detector array of 192 × 128 pixels. Optical data were visualized as different fluorescence signals and divided by a reference image acquired automatically at the beginning of each trial to produce fractional fluorescence (ΔF/F) data for all analyses and figures. Briefly, the intensity of the optical signals was measured as the ratio of a change in the fluorescence intensity (ΔF) to the initial fluorescence intensity (F), and was expressed as the percent fractional change (ΔF/F × 100). Activated area was analyzed for each color image, which was converted to a mono-color image. The converted area of each captured image was assessed as the percentage of activated area over the entire captured area (activated area/whole captured area × 100). The optical intensity and activated area were analyzed by BV Analyze (Brainvision Inc., Tokyo, Japan) and MetaMorph (Universal Imaging Co., Downingtown, PA, USA) software.

### Data analysis

Values are presented as mean ± SEM. One-way ANOVA followed by Scheffe's *post-hoc* multiple comparisons test was conducted to determine differences between groups in the behavioral test. For the optical data, differences between paired samples were analyzed using independent *t*-tests. *P* < 0.05 were considered statistically significant.

## Results

### Behavioral test

**Figure 1:**
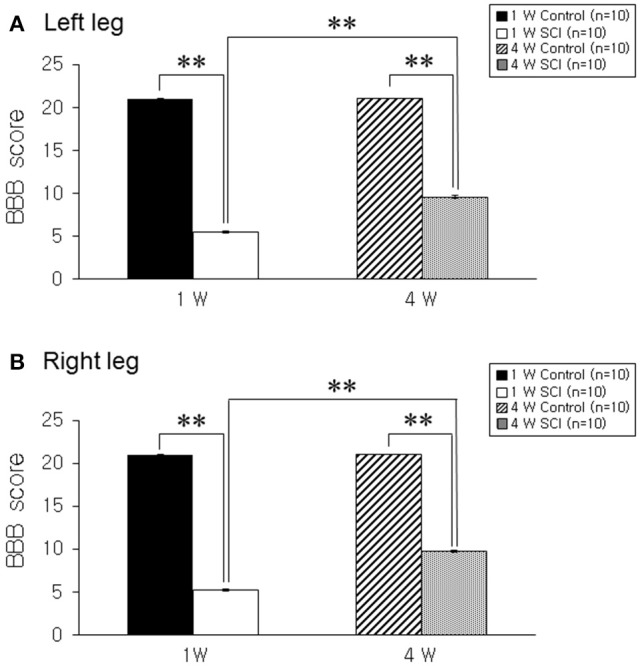
**Behavioral test. (A)** Left leg. **(B)** Right leg. The hind limb movement in 1-W and 4-W SCI rats was significantly impaired as compared to age-matched controls. The 4-W SCI group showed gradual functional recovery compared to the 1-W SCI group in both legs. Each value represents the mean ± S.E.M. ^**^p < 0.01.

The BBB score was 5.3 ± 0.17 in the left leg and 5.2 ± 0.13 in the right leg at 1 week after contusion injury. BBB scores gradually improved to 9.6 ± 0.16 and 9.7 ± 0.15 in left and right legs, respectively, at 4 weeks after SCI (Figures 1A,B). An extended recovery period did not contribute to additional improvement in BBB score (data not shown).

### Optical responses at 1 W SCI

**Figure 2:**
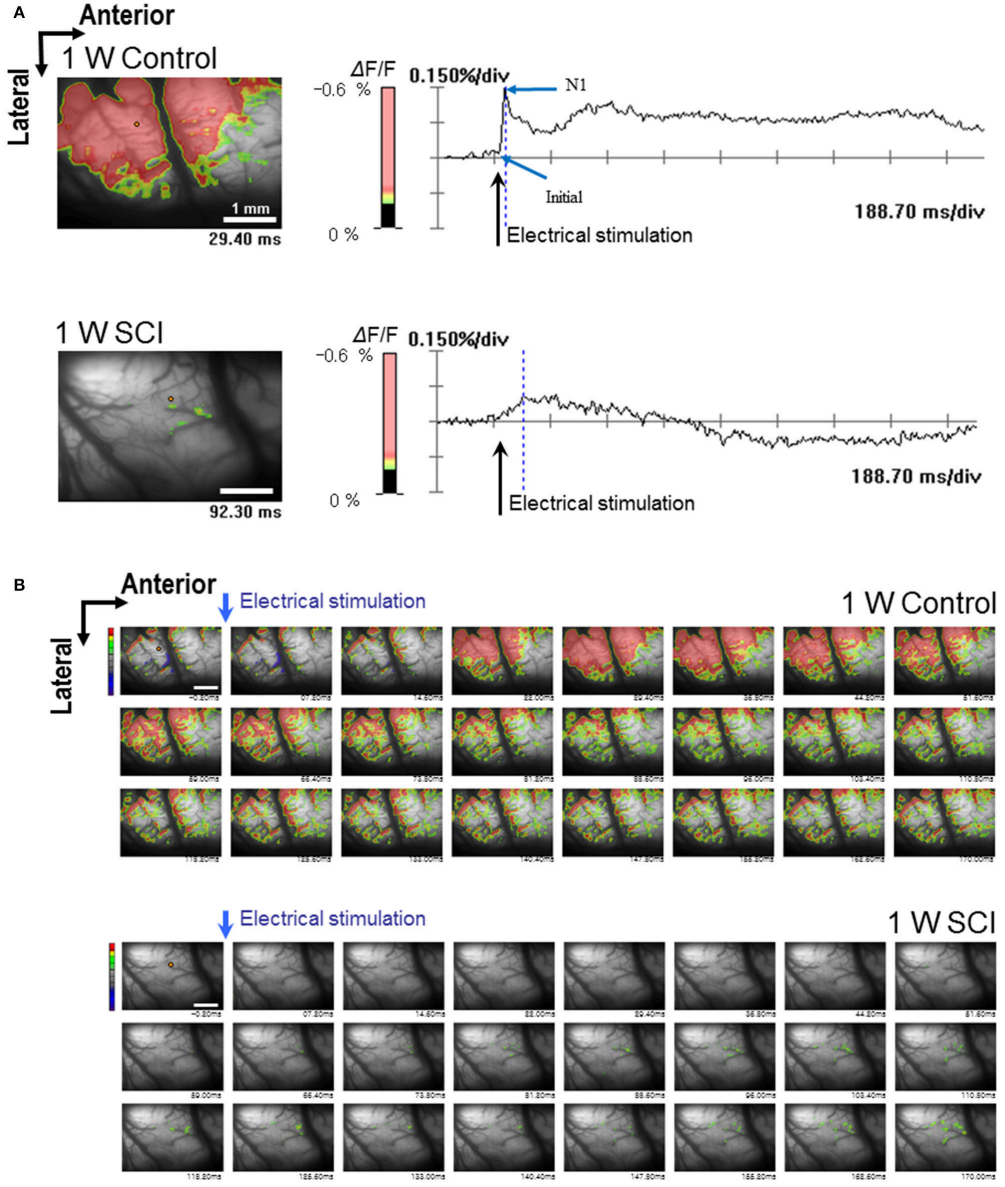
**Optical responses at 1 W after SCI**. The activated motor cortex in response to 6 mA stimulation of the L1 spinal cord is color-coded (scale bar: 1 mm). The percent change in light intensity (% ΔF/F) is displayed for optical imaging. **(A)** Wave-forms of optical signals in control (upper) and SCI (lower) rats (arrows: the time of electrical stimulation of the L1 spinal cord). **(B)** The changes in optical signals in spatiotemporal patterns by sequential imaging in control (upper) and SCI (lower) rats.

**Figure 3:**
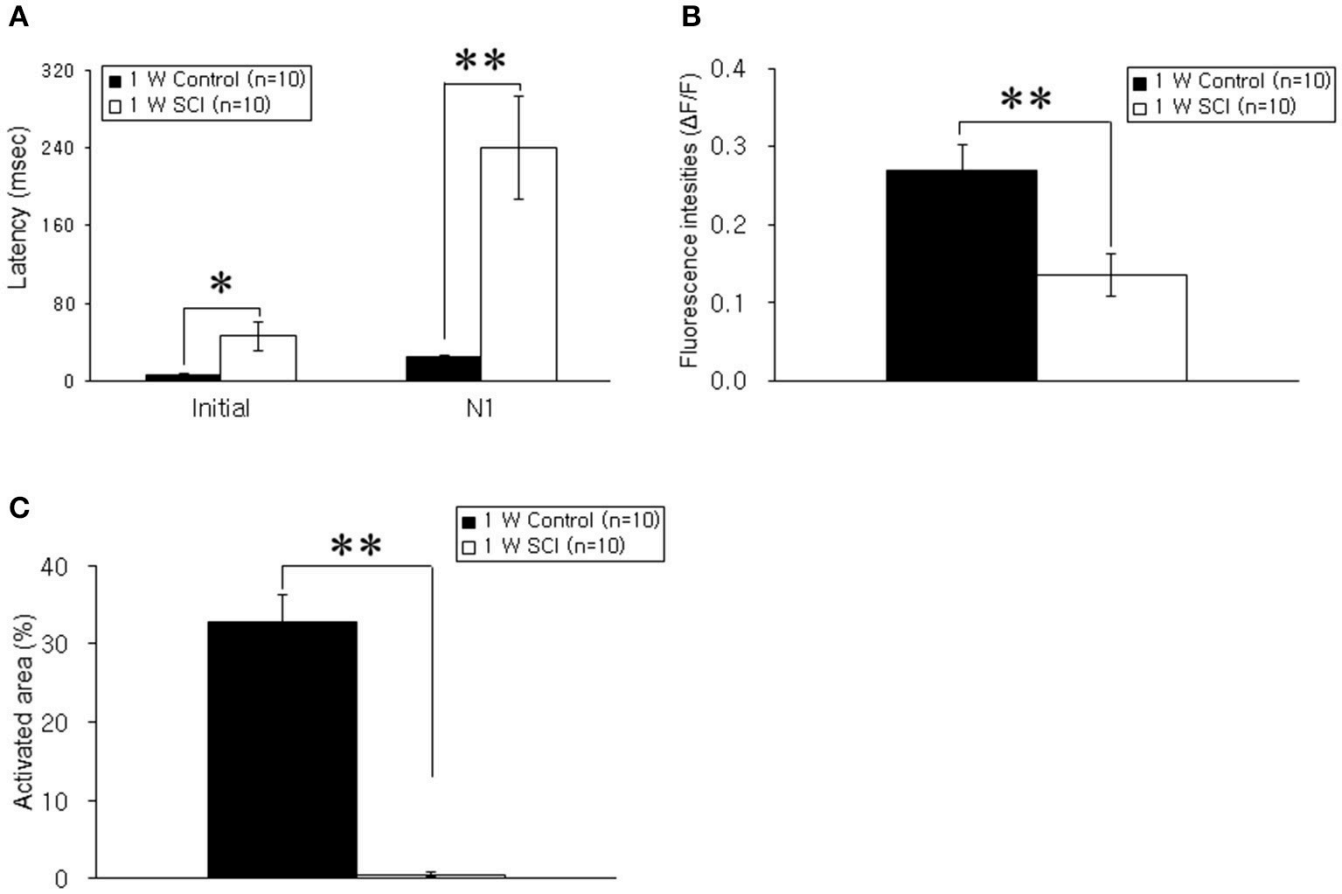
**Comparison of latency, fluorescence intensity, and activated areas at 1 week postoperatively. (A)** Latency. The latencies of optical signals in the 1-W SCI group were significantly delayed in the initial rising phase and N1 peak than those in 1-W control group. **(B)** Fluorescence intensity. The fluorescence intensity of optical signals in the 1-W SCI rats was significantly lower than that in the 1-W controls. **(C)** Activated areas. The activated area of optical signals in the 1-W SCI rats was significantly smaller than the activated area in the 1-W controls. Each value represents the mean± S.E.M. ^*^p < 0.05, ^**^p < 0.01.

Optical responses were observed in the motor cortex with different electrical stimulation intensities. Representative spatiotemporal activity patterns were recorded from the motor cortex antidromically activated by 6-mA stimulation of the ventral horn at L1 spinal cord below the injury in the 1-W control and SCI rats (Figure 2A). The initial rising phase and negative peak (N1) were distinctively observed, and activation patterns of the MEP were shown as an enlarged area after stimulation in the 1-W controls. However, at 1 week after SCI, the distribution of the activation area was diminished, and optical responses were delayed in the MEP recording (Figure 2B). In the 1-W control group, the initial rising phase and N1 latencies were 6.83 ± 0.97 and 24.59 ± 1.18 ms, respectively, upon 6-mA stimulation. Meanwhile, the latencies in the 1-W SCI group were significantly delayed in the initial rising phase (46.42 ± 13.90 ms) and N1 peak (239.6 ± 50.21 ms) than those in 1-W control group (*p* < 0.01, Figure 3A). The MEP fluorescence intensity (ΔF/F) in the 1-W SCI group was significantly lower than that in the 1-W controls (*p* < 0.01, Figure 3B). The activated area of the MEP in the 1-W SCI group (0.60 ± 0.28%) also was significantly smaller than the activated area in the 1-W control group (32.72 ± 3.40%) following stimulation (*p* < 0.01, Figure 3C).

### Optical responses in the 4 W SCI group

**Figure 4:**
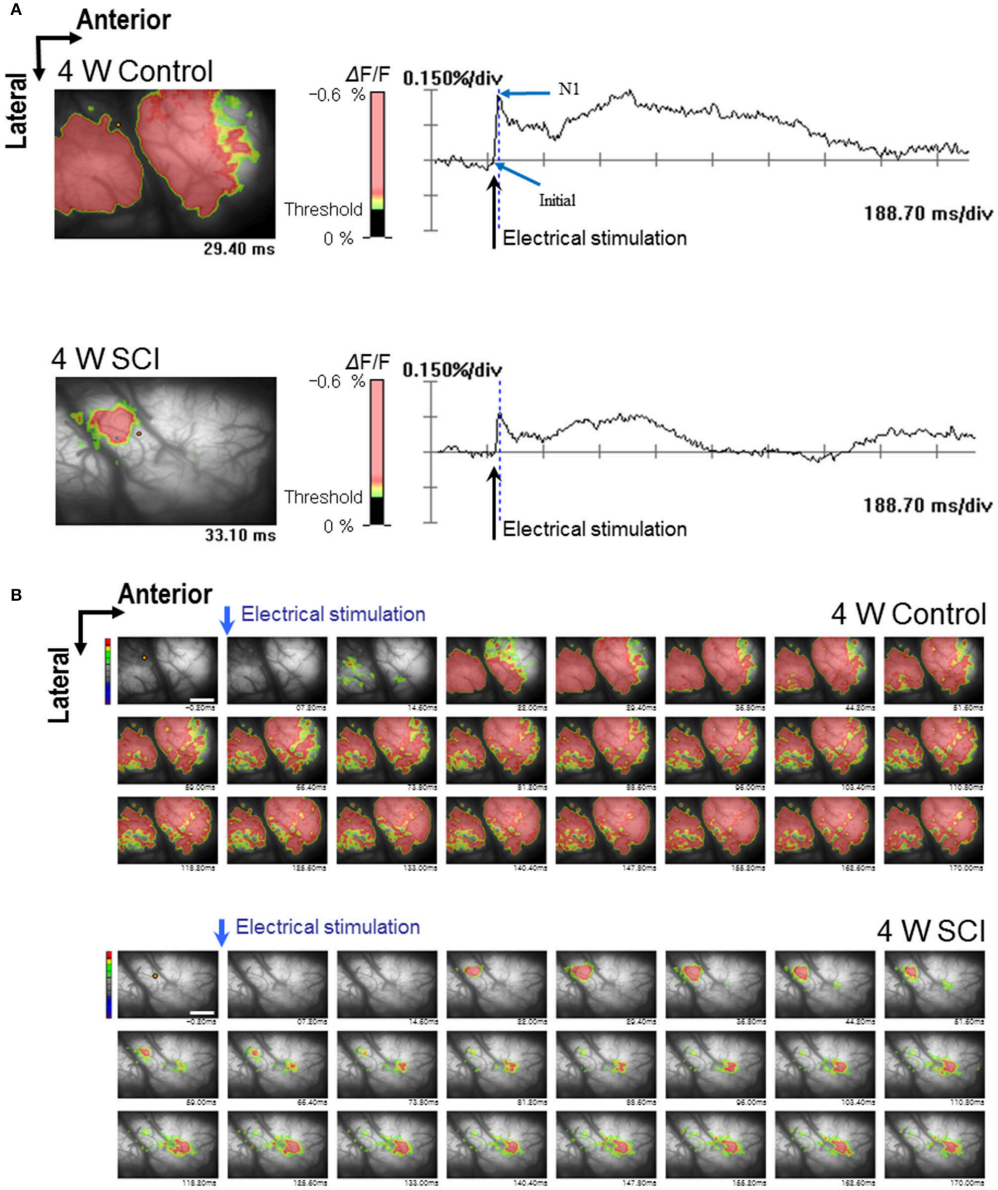
**Optical responses at 4 W after SCI**. Representative patterns of optical responses were recorded from the motor cortex following electrical stimulation of L1 spinal cord. **(A)** Wave-forms of optical signals in control (upper) and SCI (lower) rats. **(B)** Sequential images of spatiotemporal patterns of optical responses during the entire recording time in control (upper) and SCI (lower) rats.

**Figure 5:**
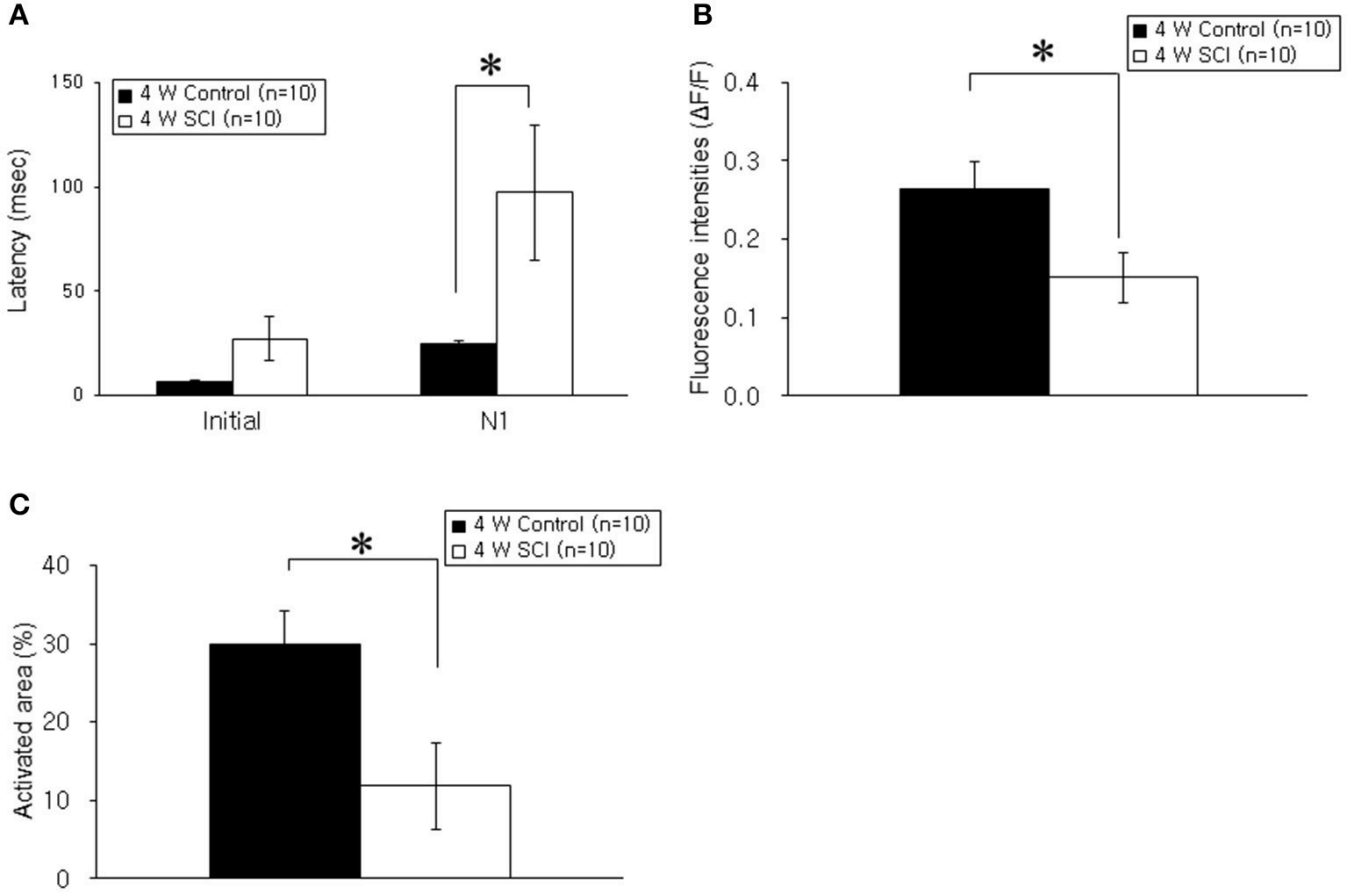
**Comparison of latency, fluorescence intensity, and activated areas at 4 weeks postoperatively. (A)** Latency. The latencies of optical signals in the 4-W SCI group were significantly delayed in the initial rising phase and N1 peak compared to those in 4-W control group. **(B)** Fluorescence intensity. The fluorescence intensity of optical signals in the 4-W SCI rats was significantly reduced compared to that in the 4-W controls. **(C)** Activated areas. The activated area of optical signals in the 4-W SCI rats was significantly smaller than that in the 4-W controls. Each value represents the mean ± S.E.M. ^*^p < 0.05.

In the optical MEP recordings at 4 weeks, the optical wave forms were more clearly shown at the initial rising phase and negative peak (N1) in the 4-W control group than in the 4-W SCI group (Figure 4A). The activated time and area were different between 4-W control and SCI groups (Figure 4B). In the 4-W controls, the initial rising phase and N1 latency were 6.46 ± 0.88 ms and 24.60 ± 1.18 ms, respectively, after 6-mA electrical stimulation. However, in the 4-W SCI group, the initial rising phase and N1 latencies were significantly delayed at 27.18 ± 9.88 and 97.11 ± 30.90 ms, respectively (*p* < 0.05, Figure 5A). The optical intensity of the 4-W SCI group was significantly reduced, compared to the 4-W controls (*p* < 0.05, Figure 5B). The activated area in the 4-W SCI group (11.83 ± 5.20%) was significantly reduced, compared to the 4-W controls (29.98 ± 4.09%) in Figure 5C (*p* < 0.05).

Within the SCI groups, the latencies in the 4-W SCI group tended to be faster than those in the 1-W SCI group (Figures 3A, 5A). Specifically, the N1 peak latency in the 4-W SCI group was significantly shorter than that in the 1-W SCI group (*p* < 0.05). The MEP fluorescence intensity in the 4-W SCI group tended to increase, but was not significantly different from that in the 1-W SCI group (Figures 3B, 5B), although the activated area in the 4-W SCI group was more widely distributed and persistent, compared to the 1-W SCI group, in MEP optical recordings (Figures 3C, 5C, *p* < 0.05).

### Stripe analysis of optical activity following electrical stimulation

**Figure 6:**
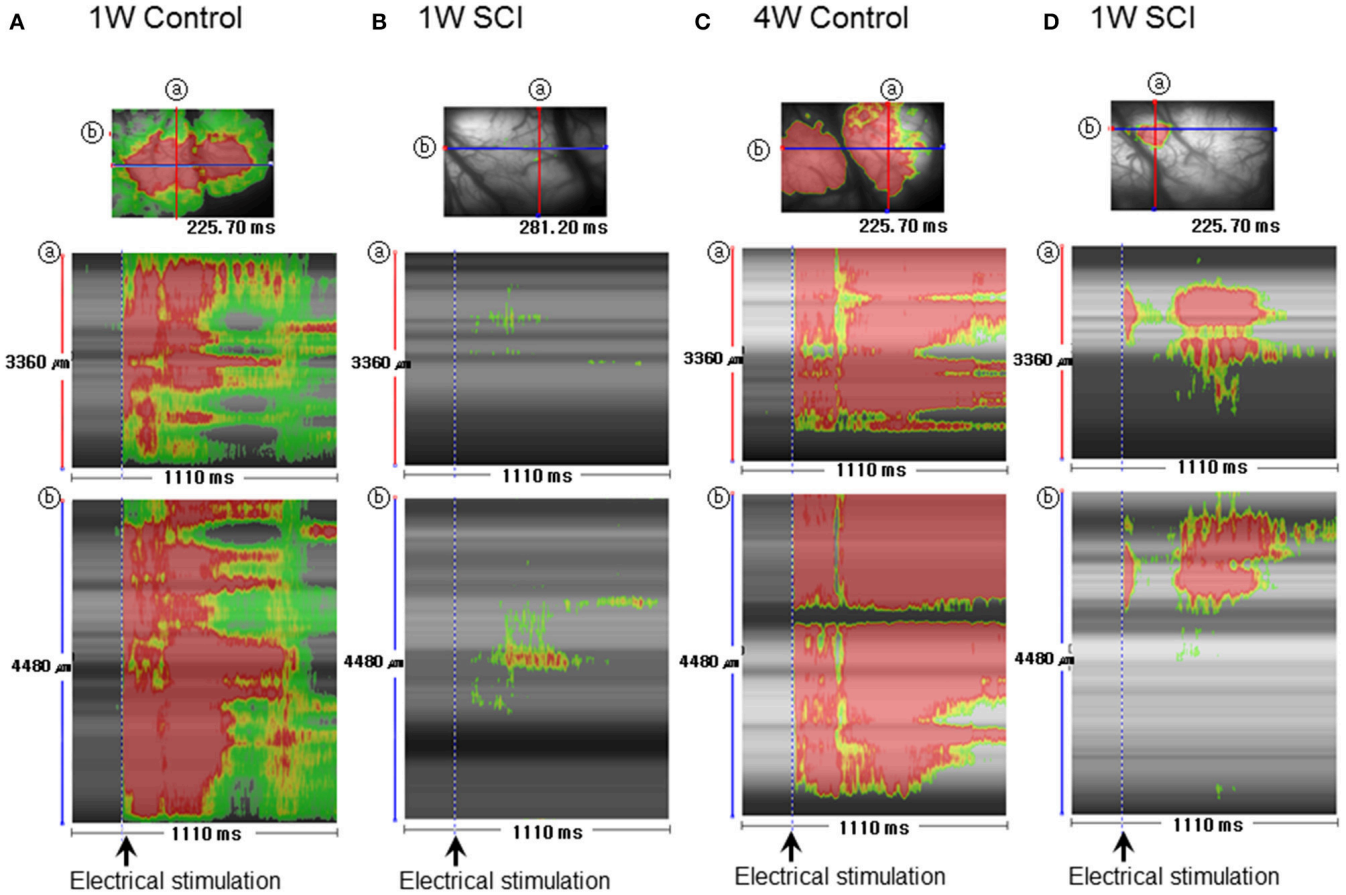
**Stripe image analysis of optical activity in the motor cortex after spinal stimulation. (A)** 1-W control. **(B)** 1-W SCI. **(C)** 4-W control. **(D)** 4-W SCI. Stripe images were obtained through the monitoring of optical activity in each group Spatiotemporal activity was obtained between 0 and 1110 ms. ⓐ and ⓑ show the spatiotemporal patterns obtained from the crossing lines in the corresponding optical images shown at the top of each column.

Striped images have the advantage of revealing temporal changes in optical signals within a specific region in different orientations. Stripe analysis of optical activity was used to evaluate spatial and temporal patterns of optical activation in the motor cortex following L1 spinal stimulation (Figure 6). The SCI groups (Figures 6B,D) showed weaker optical signals and shorter duration of the signals than the uninjured control groups (Figures 6A,C). The activation area in the 4-W SCI group (Figure 6D) was wider than that in the 1-W SCI group (Figure 6B), which was distributed and scattered. The latency of activation in the 4-W SCI group (Figure 6D) was notably shorter than that in the 1-W SCI group (Figure 6D). The optical signal activation time in the 4-W SCI group (Figure 6D) also lasted longer than that in the 1-W SCI group (Figure 6B). These results indicated that the latency was shorter and the activation duration was longer in the MEP recordings at 4-W after SCI. Therefore, the spatiotemporal pattern of optical signals in the motor cortex was deemed to differ depending on functional recovery.

### Changes in optical activation over time

**Figure 7:**
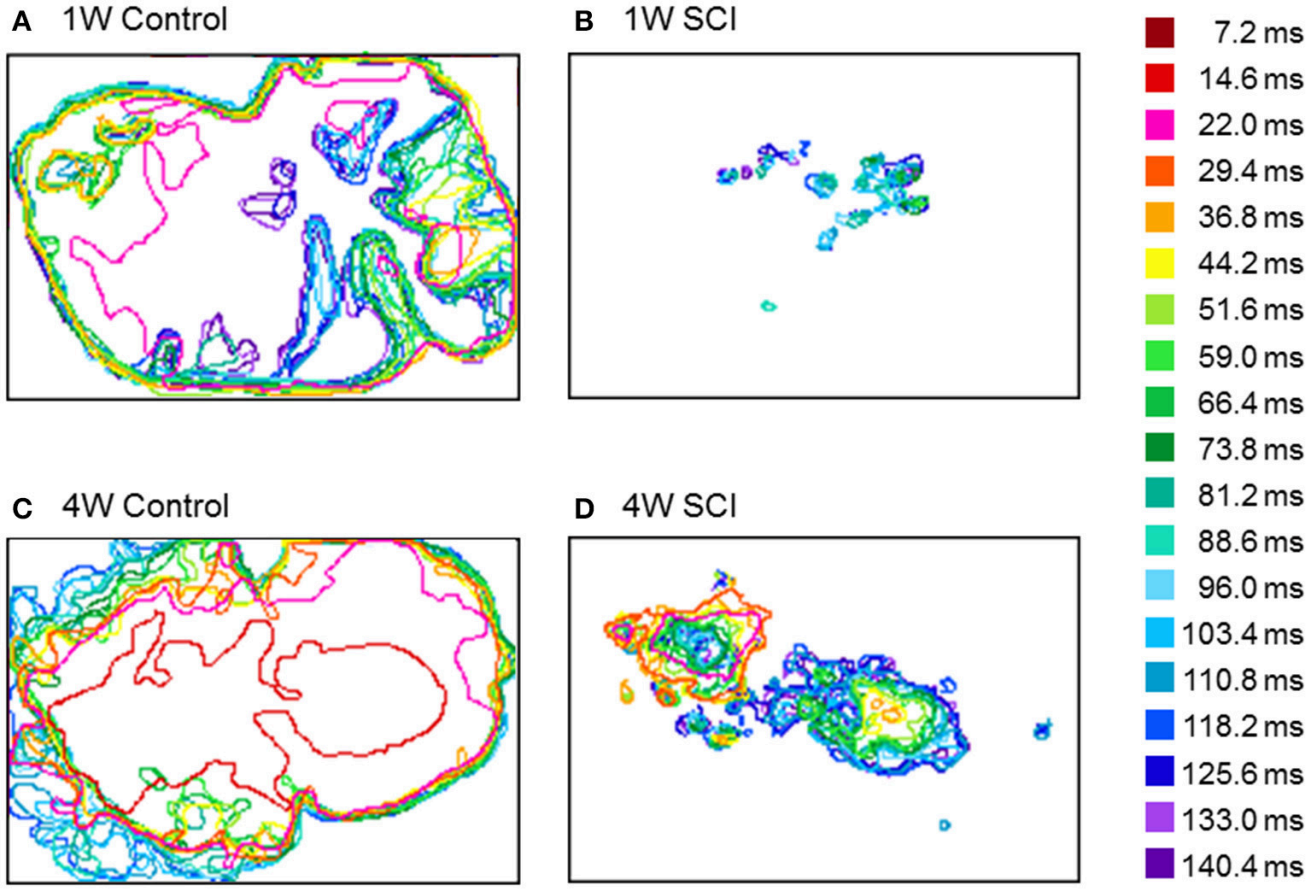
**Changes in optical activation over time. (A)** 1-W Control. **(B)** 1-W SCI. **(C)** 4-W Control. **(D)** 4-W SCI. Different colors indicate the different time frames of the optical signals after stimulation of the level below the injury site. In the control rats, optical signals after electrical stimulation were expressed over a wide cortical area at the earlier activation time **(A,C)**. However, the SCI groups showed smaller and more scattered activation areas and delayed activation time **(B,D)**. In the 4-W SCI group **(D)**, optical activation was observed earlier with less scattered activation patterns than the 1-W SCI group **(B)**.

To observe the propagation pattern by time lapse, the temporal changes in optical activity during stimulation were characterized (Figure 7). In Figure 7, different colors indicate the different time frame of the optical image. In the control rats, optical signals after electrical stimulation were expressed over a wide cortical area at an earlier activation time (Figures 7A,C). However, the SCI groups showed smaller and weaker activation areas and delayed activation time (Figures 7B,D). In the 4-W SCI group (Figure 7D), optical activation was observed earlier with less diminished activation patterns than the 1-W SCI group (Figure 7B). These suggest that the activation rapidly spreads from the focal area, depending on the condition of the spinal cord, via reorganization and/or plasticity of surviving neurons after SCI.

## Discussion

SCI induces cellular and molecular events via both primary and secondary injury cascades (Davis et al., 1996; Alonso-Calvino et al., 2016). This cascade process leads to inflammatory reactions, neuronal apoptosis, and reactive astrogliosis, which may cause impaired regeneration, tissue loss, and functional disabilities (Chen et al., 2016). In the present study, we used a moderate SCI model characterized by gradual recovery of function and lower mortality. Previous studies have shown that pluripotent endogenous neural stem cells that proliferate by damage stimulation can be isolated from the rat spinal cord (Weiss et al., 1996; Okano et al., 2007). Nestin, which is expressed during neuronal differentiation, was found to peak in ependymal cells at an adjacent injury site at 1 W SCI and to significantly decrease at 4 W post-SCI (Shibuya et al., 2002; He et al., 2017). Neuronal regeneration was visualized by labeling with NF-200 and BrdU. NF-200 is a neurofilament protein that provides a biological marker for neuronal cells, and BrdU marks cell proliferation. Both markers were present around injury sites at 2 W SCI and absent at 4 and 8 W SCI (He and Nan, 2016). Thus, most neuronal regeneration has been found to occur within 1 week after SCI and to continue for up to 4 weeks in endogenous environments. In the present study, we also observed impaired movement after SCI and gradual functional recovery at 4-weeks post-SCI via behavior tests and electrophysiological measurements.

In the present study, moderate contusion SCI resulted in the loss of descending inputs into the voluntary and automated locomotor circuitry of the spinal cord and led to partial paralysis below the injury level. Motor neuron excitability around an injury site has been found to increase over time via a number of adaptations of the spinal cord circuitry (Harvey et al., 2006; Murray et al., 2010) and to be associated with improvements in locomotor function (Fouad et al., 2010; Murray et al., 2010). However, other studies have suggested that impaired control in supraspinal centers leads to a lack of appropriate systemic modulation by inhibitory circuitry (Rekling et al., 2000; Jankowska and Hammar, 2002; Nielsen et al., 2007) and induces maladaptive plasticity in neurons below the level of injury (Boulenguez et al., 2010).

MEPs are a useful method for investigating changes in the motor neuron properties and in spinal and descending modulatory actions following injury and recovery (Cao et al., 2005; Diehl et al., 2006). A positive correlation has been shown between the MEP amplitude and the amount of preserved spinal cord tissue at the site of injury (Garcia-Alias et al., 2003). Conduction tests can be applied to the injured nerve by stimulating at one site and recording at another distant site. Therefore, MEPs could be used to assess the severity of functional deficits caused by damage of myelinated fibers and to reflect the physiological functions of the remaining and/or reorganizing neuronal synaptic networks. Somatosensory evoked potentials (SSEPs) are typically recorded from the somatosensory cortex following stimulation of peripheral nerves, while MEPs are elicited by stimulation of the brain and recorded from muscles. Transcranial stimulation applied over the skull activates cortical and subcortical (brainstem) descending motor pathways (Adamson et al., 1989; Schlag et al., 2001) that connect with spinal interneurons, which in turn activate the motor neurons. The normal direction of propagation in an axon is called the orthodromic direction; an action potential propagating in the opposite direction follows an antidromic direction (Haghighi et al., 1994). Previous studies showed that once the corticospinal tract of the spinal cord is electrically stimulated, its antidromic activity can be recorded from the cerebral cortex (Humphrey, 1968a,b; Partanen et al., 2000). In the present study, we applied antidromic stimulation to observe spatial and temporal changes of MEP within the brain. In rat brain, known as an amalgam between sensory/motor function, the sensory and motor representations overlap partially (Donoghue and Wise, 1982). We also observed that some optically activity regions of SSEP and MEP stimulation overlap; these areas comprised a very large portion (50%) of the total active area (data not shown). However, our optical recording results agreed well with those of Holthoff et al. (2007), who reported that intrinsic optical signals in brain slices can be used to evaluate functional connectivity. We were also able to observe changes in the brain by optical recording in the motor cortex after spinal stimulation.

We found that the motor cortex regions displaying peak fluorescence were most distinct in the 4-W MEP recordings, even though the expression area was more restricted and smaller than that in the controls (see Figure 4). It should also be noted that some optically active regions were observed. These areas were diminished in the 1-W SCI group, wherein expression times were also significantly delayed. The delayed activating optical signals might be resulted from reduced populations of surviving neurons and increased demyelination after SCI (Alonso-Calvino et al., 2016; Chen et al., 2016). However, improved activating optical signals 4 weeks after SCI may have reflected spontaneous recovery, such as newly formed neuronal reorganization and neuroplasticity of the remaining neuronal synapses in the adjacent injury site (Murray et al., 2011; He et al., 2017). These results were consistent with the behavior test results. Thus, the enhanced activity and behavior suggested a mechanism for neural plasticity in the cortical networks altered by injury. Taken together, our findings indicated that optical recordings reveal considerable functional recovery and reconfiguration of the motor network temporally and spatially via antidromic stimulation of the corticospinal tract after SCI. In the present study, we showed that the optical signal of MEPs closely reflect the severity of spinal cord injury. Our data demonstrated that optical mapping is an efficient and consistent means to document the integrity of the corticospinal and subcortical pathways without penetration into the brain. In addition, optical recording allowed sensitive detection of spinal cord injuries of different grades over time and provided potential information about recovery.

## Conclusion

In the present study, nerve conduction in the 4-W SCI group improved spontaneously, compared to the 1-W SCI group, and was in agreement with behavioral observations. Optical signals were significantly reduced in the SCI rats, compared to the control rats. The activation area around the motor cortex was expanded and enlarged in the 4-W SCI group, compared to the 1-W SCI group. Thus, we deemed that optical imaging with VSD could allow for determination of spatiotemporal patterns and accurate characterization of MEPs. Our results further suggested that optical imaging may be useful in recording the functional changes that reflect conditions of reorganization and/or changes in surviving neurons after SCI.

## Author contributions

KL, UK, and BL designed the study. KL, UK, SP, and YP performed the experiments. KL, UK, SP, YP, and BL analyzed the data. KL, UK wrote the manuscript. KL and UK contributed equally to this work.

### Conflict of interest statement

The authors declare that the research was conducted in the absence of any commercial or financial relationships that could be construed as a potential conflict of interest.
